# Overview of therapeutic applications of non-invasive vagus nerve stimulation: a motivation for novel treatments for systemic lupus erythematosus

**DOI:** 10.1186/s42234-021-00069-5

**Published:** 2021-05-25

**Authors:** Charrise M. Ramkissoon, Amparo Güemes, Josep Vehi

**Affiliations:** 1 Institut d’Informàtica i Aplicacions, Universitat de Girona, Girona, Spain; 2 Bio-Inspired Technology, Department of Electrical and Electronic Engineering, Imperial College London, South Kensington Campus, London, UK; 3 Centro de Investigación Biomédica en Red de Diabetes y Enfermedades Metabólicas Asociadas (CIBERDEM), Madrid, Spain

**Keywords:** Systemic lupus erythematosus, Vagus nerve stimulation, Autonomic nervous system, Non-invasive, Bioelectronic medicine

## Abstract

Systemic lupus erythematosus (SLE) is a chronic systemic autoimmune disorder that commonly affects the skin, joints, kidneys, and central nervous system. Although great progress has been made over the years, patients still experience unfavorable secondary effects from medications, increased economic burden, and higher mortality rates compared to the general population. To alleviate these current problems, non-invasive, non-pharmacological interventions are being increasingly investigated. One such intervention is non-invasive vagus nerve stimulation, which promotes the upregulation of the cholinergic anti-inflammatory pathway that reduces the activation and production of pro-inflammatory cytokines and reactive oxygen species, culpable processes in autoimmune diseases such as SLE. This review first provides a background on the important contribution of the autonomic nervous system to the pathogenesis of SLE. The gross and structural anatomy of the vagus nerve and its contribution to the inflammatory response are described afterwards to provide a general understanding of the impact of stimulating the vagus nerve. Finally, an overview of current clinical applications of invasive and non-invasive vagus nerve stimulation for a variety of diseases, including those with similar symptoms to the ones in SLE, is presented and discussed. Overall, the review presents neuromodulation as a promising strategy to alleviate SLE symptoms and potentially reverse the disease.

## Background

Systemic lupus erythematosus (SLE) is a chronic systemic autoimmune disorder that commonly affects the skin, joints, kidneys, and central nervous system.

SLE pathogenesis consists of a hyperactivation of the immune response, which is characterized by a drastic increase in the levels of pro-inflammatory cytokines and their receptors in target organs (Davis et al. 2011). Another key component in the pathogenesis of SLE is the elaboration of anti-DNA and related antinuclear autoantibodies such as T cell-dependent B cell autoantibody production. This network of autoantibodies and cytokines elaborated by both the innate and the adaptive immune systems that activate and facilitate the interaction between B and T cells is at the heart of the exacerbated inflammatory response observed in SLE (Pacheco et al. 2017). Several cytokines have been implicated in SLE pathogenesis, including interferon- *α* (IFN- *α*), tumor necrosis factor (TNF), and interleukins (IL-1, IL-6, IL-10, IL-17, IL-21). These cytokines are also key drivers for other autoimmune diseases like rheumatoid arthritis, so it is not surprising that these two diseases share similar clinical manifestation and therefore current treatments are being similarly developed to target them.

Many patients experience bursts of autoantibodies against nuclear components interspersed with quiescent periods. SLE is heterogeneous in nature and therefore, patients can experience a wide range of symptoms with varying severity (Dörner and Furie 2019). The current understanding of the factors that drive the different phenotypes and its pathogenesis in SLE is limited and directly affects the treatment of patients. The current therapeutic approach to SLE most commonly includes the administration of antimalarials, glucocorticoids, immunosuppressants, and biological agents. These treatments have greatly improved the clinical scenario, with a significant reduction of mortality to 10% within 10 years, compared to 50% within 3 years in the 1960s as shown by an analysis of a multisite international SLE cohort (Bernatsky et al. 2006). However, current medication options produce adverse secondary effects. Anti-malarial agents have been associated with ocular toxicity, which increases with age, and renal damage (Marmor et al. 2016). Immunosuppressant use is associated with risks of infections, hematological toxicities, gastrointestinal events, and ovarian toxicities. (Oglesby et al. 2013). Glucocorticoids, irrespectively of the route or formula of glucocorticoid administration, are also related with long-term adverse effects on musculoskeletal, cardiovascular, peripheral vascular, ocular, and metabolic domains that have been associated with significant comorbidity (Al Sawah et al. 2015). Additionally, immunological mechanisms accounting at least in part for the occurrence of suboptimal response to glucocorticoids in SLE have been proposed (Guiducci et al. 2010). Finally, Belimumab, the only biological agent that is FDA approved for the treatment of SLE, has been associated with arthralgia, nausea, headache, and infections, including bacterial upper respiratory tract infection, viral upper respiratory tract infection, and bacterial urinary tract infection (Furie et al. 2018).

Furthermore, SLE patients still experience an increased mortality rate of 2- to 5-fold compared with the general population (Nossent et al. 2007), with particular risk associated with the female sex, younger age groups, fewer years with SLE diagnosis, and black ethnic groups. This directly translates to an increased economic burden on both patients and healthcare systems. Several studies (Kariburyo et al. 2020; Román Ivorra et al. 2019) found that medications, inpatient stays, laboratory investigations, day hospitalizations, biopsies/imaging tests, and specialist visits drive up costs. Severe flares, active renal disease, and organ damage were identified as major independent cost predictors. Indirect costs accounted for absenteeism due to sick leave, and short-term and long-term work disability. Patient quality of life was found to be related to age, disease activity, organ damage, and severity.

To reduce mortality, decrease economic burden, and alleviate side effects of current pharmacological interventions used to treat SLE, other treatment modalities must be explored. The use of non-pharmacological means to treat autoimmune diseases has recently spiked the interest of investigators. Of these therapies, vagus nerve stimulation (VNS) has shown much promise (Fox 2017). VNS promotes the upregulation of the cholinergic anti-inflammatory pathway (CAP), which reduces the activation and production of proinflammatory cytokines and reactive oxygen species, culpable processes in autoimmune diseases (Kenney and Ganta 2014). This article will perform a deep dive into the molecular circuitry involved in this response driven by VNS and the application of non-invasive VNS (nVNS) to treat inflammatory disorders such as SLE. This will include a brief explanation of autonomic nervous system (ANS) dysregulation in SLE, the anatomy of the VN, a brief history of VNS, and a review on nVNS. It is worth highlighting that the symptoms in SLE can mimic those of other autoimmune diseases, infectious diseases, endocrine abnormalities, chronic fatigue, and fibromyalgia (Cojocaru et al. 2011). In fact, the link between these aforementioned diseases and disorders produces a 25% chance that patients with one autoimmune will develop additional autoimmune disorders (Mohan and Ramesh 2003). As a result, therapies developed to improve the clinical outcomes of these diseases, such as VNS, are also of interest for SLE and some remarkable clinical trials have therefore been included in this review. The article will be concluded with a section dedicated to the outlook of the use of nVNS for SLE treatment.

## Systemic lupus erythematosus and the autonomic nervous system

The ANS, composed of two primary branches, the sympathetic nervous system (SNS) and the PNS, plays a critical role in mediating interactions between the nervous and immune systems. It coordinates the interplay among cells, tissues, and organs throughout the body to maintain homeostasis via a widespread innervation of glands, smooth muscles, and the heart (Capellino et al. 2008). Increased sympathetic nerve outflow related to the SNS has been shown to activate pro-inflammatory cytokines and produce reactive oxygen intermediates (Kenney and Ganta 2014), which are strongly implicated in the pathogenesis of SLE as previously described. SLE is found to have SNS predominance or PNS dysregulation, as reflected by decreased heart rate variability in patients with SLE (Poliwczak et al. 2018; Matusik et al. 2018). The prevalence of autonomic dysfunction ranges widely from 6 to 93% in patients with SLE (Milovanović et al. 2010). This autonomic imbalance is related to an increased risk of developing cardiovascular disease, which is a major cause of morbidity and mortality in patients with SLE (Malpas 2010). Recent studies suggest that vagus nerve stimulation may be a strategy to reverse the autonomic imbalance (Gurel et al. 2020) aiming to ameliorate, and potentially suppress, the development of this disease. Furthering this idea, the following sections provide an overview of the vagus nerve anatomy and it’s contribution to the anti-inflammatory pathways.

## The vagus nerve

**Fig. 1:**
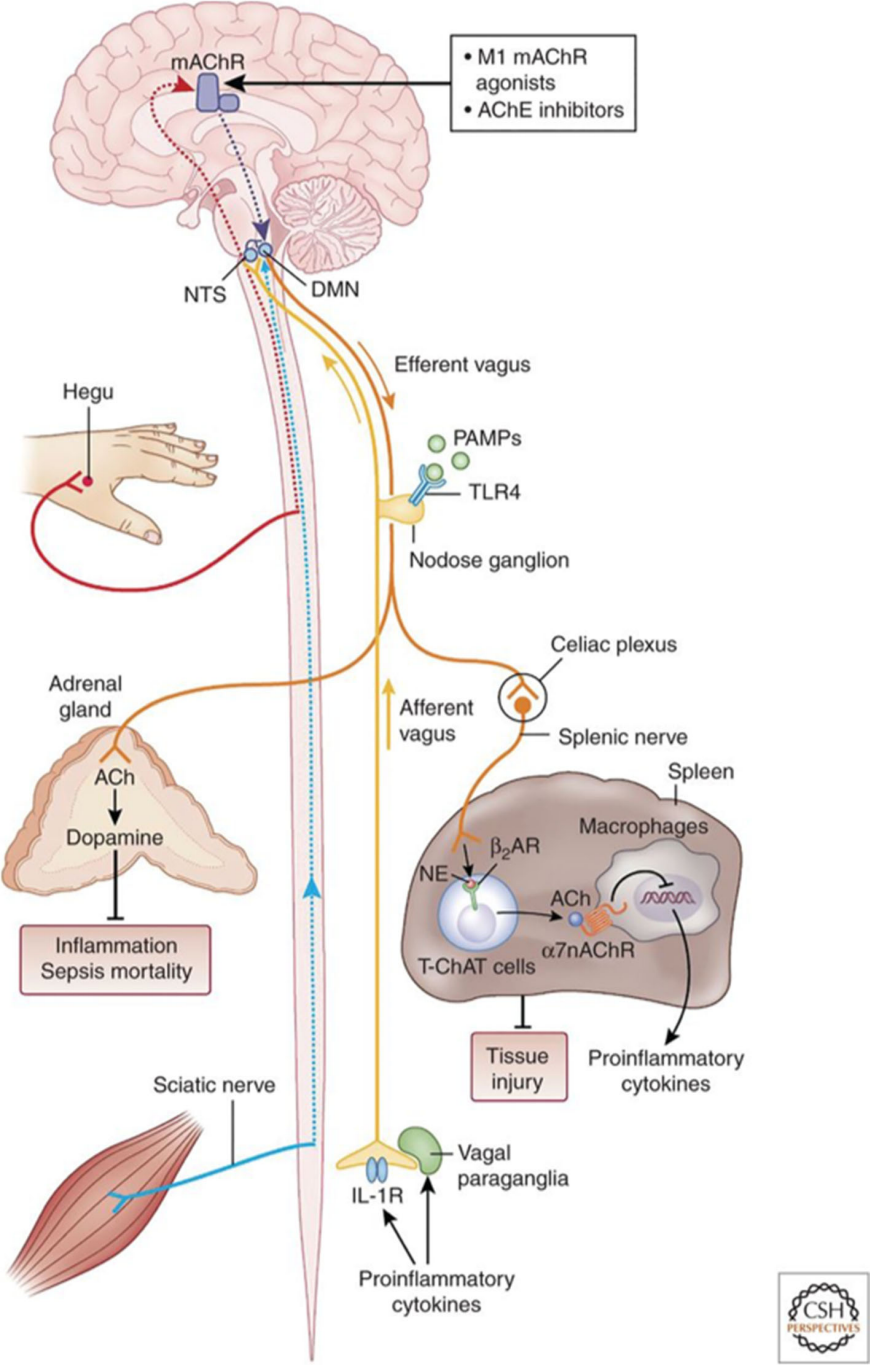
The Vagus Nerve and the Inflammatory Reflex (Pavlov et al. 2020)

The vagus nerve (VN) depicted in Fig. 1 is the tenth cranial nerve and is the longest nerve of the organism, which links the central nervous system and the body by innervating major visceral organs such as the heart, the lungs, and the gastrointestinal tract. The VN is a mixed nerve with 20% efferent and 80% afferent fibers (Prechtl and Powley 1990), and is a major component of the PNS. It therefore enables bi-directional communication between the brain and the different organs of the body, transmitting both sensory and motor information and acting as a primary conduit for bi-directional information exchange.

The VN emerges from or converges onto four main nuclei of the brainstem, which include the dorsal motor nucleus (DMN) of the VN, that innervate the intramural ganglia associated with thoracic and abdominal viscera (heart, the lungs, and the gastrointestinal tract), the nucleus ambiguus, which gives rise to the branchial efferent motor fibres and preganglionic neurones innervating the heart, the solitary nucleus, which conveys afferent fibres from visceral organs, and the spinal trigeminal nucleus, which receives sensory information from the outer ear, and the mucosa of the larynx, among others (Thompson et al. 2019).

From its origin in the brain, the VN runs caudal to the glossopharyngeal nerve and superficial to the internal jugular vein towards the jugular foramen. Within and below to the jugular foramen lie two ganglia associated with the VN, the superior (jugular) and inferior (nodose) ganglia, respectively (Câmara and Griessenauer 2015). Motor and sensory fibres pass through the jugular ganglion, and only some visceral afferent fibres have cell bodies in the nodose ganglion, which primarily relays information from the pharynx and thoracic and abdominal viscera (Câmara and Griessenauer 2015). At the cervical level, the VN continues its descent travelling between the internal jugular vein and the internal and external carotid arteries, and bifurcates into different branches innervating the larynx, bronchi, lungs, heart, and esophagus (Johnson and Wilson 2018). In the lower esophagus, the left vagus runs ventrally whereas the right bundle runs dorsally. After crossing the diaphragm, the dorsal (or posterior) subdiaphragmatic vagus branches into the dorsal gastric branch, and the dorsal celiac branches. On the other hand, the ventral subdiaphragmatic vagus divides into the ventral (or anterior) gastric and celiac branches, and the common hepatic branch. Both the ventral and dorsal gastric branches innervate the stomach and proximal duodenum, whereas the celiac branches innervate the distal duodenum and colon. The hepatic branch further bifurcates into the main hepatic branch that innervates the liver, and the gastroduodenal branch, that innervates the duodenum and proximal pancreas (Johnson and Wilson 2018).

Vagal fibers can be described according to the classification by Erlanger and Gasser (1937) (Erlanger and Gasser 1937) based on their nerve conduction properties in A, B and C fibers (see Table 1). Each type of fibre carries different physiological information, with large myelinated A-fibers mostly convey somatic afferent and efferent signals, small myelinated A-fibers visceral transmit afferent signals, B-fibers provide efferent sympathetic and parasympathetic pre-ganglionic innervation, and small unmyelinated C-fibers comprise most of the afferent visceral innervation.

**Table 1:** Characterization of vagus nerve fibers.

Type of fiber	Size (diameter)	Myelinated	velocity range (*ms*)	Transmitted information
A-fiber	Large (5-20 *μ*m)	Yes	>4.5	Afferent visceral information and efferent motor signals
B-fiber	Mid-size (1-3 *μ*m)	Yes	2-4.5	Most of the efferent parasympathetic signals
C-fiber	Small (0.4-2 *μ*m)	Yes	<2	Mainly afferent visceral information

Their size, and therefore conduction properties, affect the excitation thresholds at which they respond to electrical stimulation. A-fibers have the lowest threshold followed by B- and C-fibers (Stauss 2017). The stimulation threshold is determined by the total charge delivered to the nerve fiber, which depends on the stimulation current, the pulse duration, the stimulation frequency (i.e. the rate at which these pulses are applied) and the waveform, among others (Stauss 2017). However, a study suggests that the threshold is not dependant on the polarity of the electrode, at least within the specific range of stimulation parameters used in the analysis (pulse durations of 0.05-20 ms; frequencies of 2-20 Hz; amplitudes of 3V and 6V) (Stauss 2017). In general, higher stimulation currents, longer pulse durations, and/or higher stimulation frequencies are needed to activate smaller nerve fibers. As a result, A-fibers are recruited first with the lowest stimulation current (0.01–0.2mA), followed by B- (0.04–0.6 mA) and C-fibers (>2.0mA) with increasing stimulation intensities. Consequently, the physiologic response to VNS greatly depends on the recruitment of fibres with the different stimulation parameters.

### Anti-inflammatory pathways

There are two anti-inflammatory pathways of interest for VNS that interface between the nervous and immune systems: the hypothalamus-pituitary-adrenal (HPA) axis and the cholinergic anti-inflammatory pathway (CAP). The HPA axis is involved in coordinated neural, behavioral, and endocrine responses that provide an important first-line innate defense against infection and inflammation and help to restore homeostasis in the body (Johnson and Wilson 2018). The vagal afferent fibres located at the paraganglia level are equipped with receptors for pro-inflammatory cytokines, interleukin (IL)-1 *β*, that when engaged activate a cascade reaction that leads to the release of glucocorticoids by the adrenal glands to decrease peripheral inflammation via the HPA axis (Bonaz et al. 2016). The HPA axis is also activated by circulating pro-inflammatory cytokines on circumventricular organs (Buller 2001).

The CAP on the other hand is mediated through vagal efferent fibers that originate at the DMN, as it has been recently found (Kressel et al. 2020), ultimately resulting in the inhibition of the release of pro-inflammatory cytokines such as tumor-necrosis factor-alpha (TNF)- *α* in the liver, spleen, and heart, and the attenuation of serum concentrations of TNF- *α* (Borovikova et al. 2000). Interestingly, although the inflammatory reflex is mainly driven by the VN, there is no evidence that cholinergic parasympathetic fibers innervate the spleen or that it directly interacts with resident macrophages in the gut (Rosas-Ballina et al. 2008; Sundman and Olofsson 2014). It is considered, however, that the VN contributes in the anti-inflammatory response through a vagosympathetic synergistic effect by activating the sympathetic fibers in the splenic nerve via cholinergic transmission at the celiac ganglia and the superior mesenteric ganglion in the celiac plexus, where the splenic nerve originates, which in turn reduces pro-inflammatory cytokine production in the spleen and the liver, and mediates the inhibition of TNF release from splenic macrophages (Kressel et al. 2020; Rosas-Ballina et al. 2008; Sundman and Olofsson 2014; Pavlov et al. 2020; Huston et al. 2006). It is worth noting that this inhibition of TNF release ultimately mediated by the vagus nerve is dependant upon nicotinic cholinergic receptors (*α*_7_nAChRs) (Olofsson et al. 2012), not muscarinic receptors as in the PNS, yet the splenic fibers do not produce ACh per se (Sundman and Olofsson 2014). Previous work on the source of this ACh in the spleen has shown that 1) some lymphocytes, such as T cells, are able to produce ACh as they contain functional choline acetyltransferase (ChAT) (Fujii et al. 1996), 2) these are found close to adrenergic nerves (Rosas-Ballina et al. 2011; Huston et al. 2006), and 3) they express *β*2-adrenergic receptors (*β*2-ARs) (Pavlov et al. 2020; Vida et al. 2011). These findings strongly suggest that lymphocytes in the spleen mediate the signalling mechanism between the adrenergic nerves releasing norepinephrine (NE) and the inhibition of systemic TNF release by macrophages through lymphocyte-synthesised ACh binding to nicotinic receptors (*α*_7_nAChRs). This complex interaction is at the core of the inflammatory reflex.

In summary, activation of the VN (e.g. via stimulation), causes the release of ACh that binds to muscarinic receptors at the celiac plexus, which in turn activates the splenic nerve. The NE released from the terminals of the splenic nerve binds to adrenergic receptors expressed in lymphocytes in the spleen, which promotes the synthesis of endogenous ACh. This ACh then binds *α* nicotinic receptors expressed in macrophages and suppresses proinflammatory cytokine release and inflammation. Despite this long sequence of interactions within this pathway, the high speed of neural conductance that characterises the CAP allows localized input to the region of inflammation (Sundman and Olofsson 2014).

## Vagus nerve stimulation

**Fig. 2:**
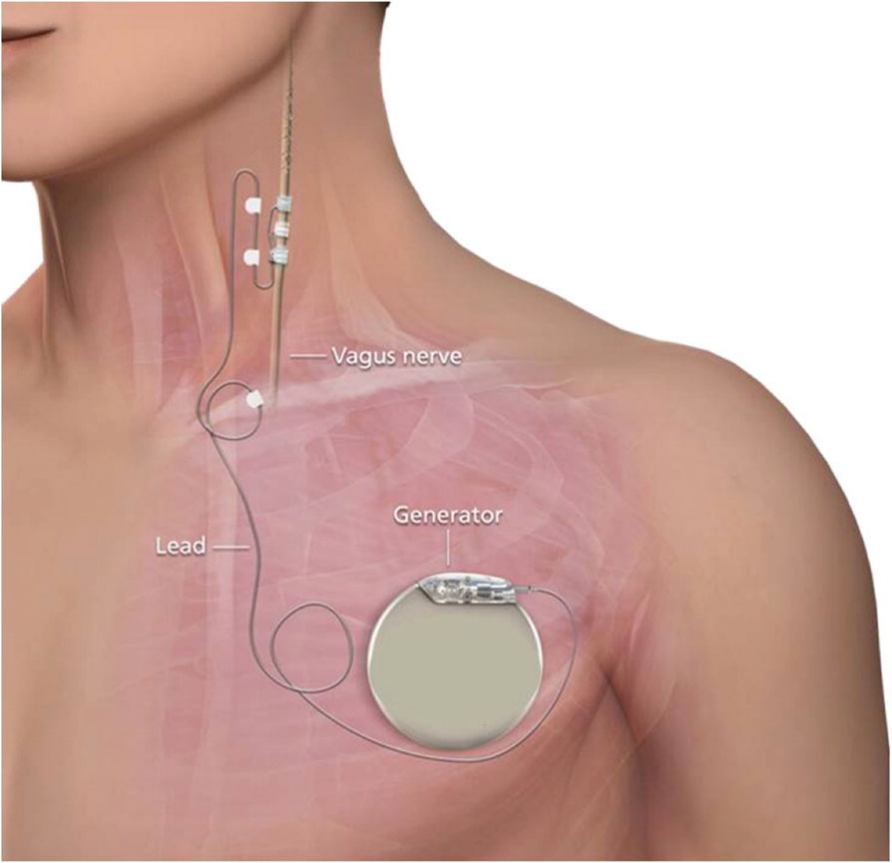
Implantable Vagus Nerve Stimulation Device (Verrier et al. 2016)

VNS, discovered in the late 19th century, was first proposed to treat epilepsy by James Corning (Lanska 2002) although his attempts were ultimately unsuccessful. Later on, experiments in animal models demonstrated the potential antiepileptic properties of VNS (Aalbers et al. 2011). These were then followed by human studies, which began in the 1990s and demonstrated a substantial decrease in seizure frequency in refractory and intractable epilepsy (Ben-Menachem et al. 1994). In 1997, an implanted cervical VNS device (NeuroCybernetic Prosthesis System, Cyberonics, Inc, Houston, TX, USA - now Livanova, Fig. 2) was approved by the US Food and Drug Administration (FDA) for use as an adjunctive treatment (with drugs or surgery) for patients over 12 years of age with medically refractory partial onset seizures (Morris et al. 2013). In 2005, the same VNS device was approved by the FDA for long-term adjunctive treatment of chronic/recurrent depression for patients over 18 years of age with a major depressive episode with inadequate responses to four or more antidepressant treatments (RH 2014). By 2013, over 100,000 VNS devices were implanted worldwide for the treatment of epilepsy in over 70,000 patients (Elliott et al. 2011) and the American Academy of Neurology guideline associates VNS with an increase in the amount of patients experiencing a ±50% reduction in the number of seizures (Morris et al. 2013).

Recent studies of VNS in in vivo systems have shown its anti-inflammatory properties which have led to more preclinical research aimed at expanding VNS treatment across a wider range of inflammatory disorders. VNS acting through the CAP as previously described has shown promising results in treating chronic inflammatory disorders such as sepsis, lung injury, rheumatoid arthritis (RA), and diabetes (Johnson and Wilson 2018; Koopman et al. 2018). It is also being used to control pain in fibromyalgia (Lange et al. 2011) and migraines (Silberstein et al. 2016). Additionally, VNS has shown to be useful in the treatment of obesity and heart disease (Johnson and Wilson 2018).

The effect of VNS in epilepsy and depression is thought to be mediated through the activation of vagal afferent fibres, performed at high frequency of stimulation (20–30 Hz) whereas, the activation of the CAP has been found to be mediated through vagal efferent fibres and involves a low-frequency (1–10 Hz) stimulation of the VN. A study by Borovikova et al. (Borovikova et al. 2000) found that VNS at 1 Hz frequency for 20 min was effective for the preferential recruitment of efferent parasympathetic fibres. Other reports have also shown that low frequency (5 Hz) VNS is able to activate vagal efferent fibres (Bernik et al. 2002). Implantable VNS devices (Cyberonics, Houston, TX, USA) in humans at varying frequencies have been found to improve outcomes in several inflammatory diseases mainly, RA (10 Hz) (Koopman et al. 2018), Crohn’s disease (10 Hz) (Bonaz et al. 2016), and fibromyalgia (20 Hz) (Lange et al. 2011), with some patients achieving disease remission in all three studies.

### Non-invasive vagus nerve stimulation

Implantation of a VNS device is associated with complications such as, bradycardia, asystole, delayed arrhythmias (although rare), syncope, cough, paraesthesia, pain, sleep apnea, surgical trauma or VN trauma with unilateral vocal cord dysfunction and dyspnoea, and thermal injury to the VN and adjacent structures due to radiofrequency exposure (Asconapé et al. 1999; Iriarte et al. 2009; Fahy 2010; Marzec et al. 2003; Handforth et al. 1998). To avoid surgical implant-related complications, researchers have developed nVNS devices. To date, two pathways of nVNS pathways exist: transcutaneous cervical (TC-VNS) and transcutaneous auricular (TA-VNS).

**Fig. 3:**
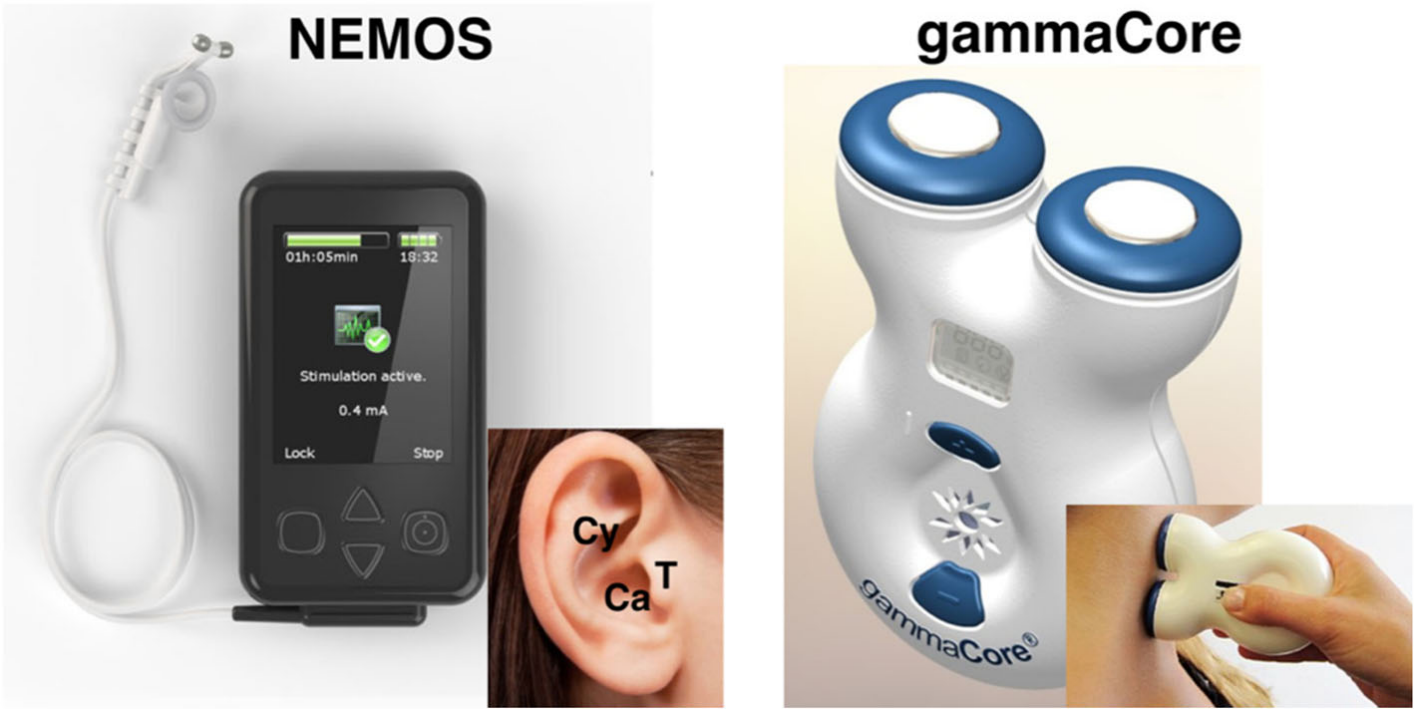
Two non-invasive VNS devices. NEMOS is a transauricular VNS device that stimulates the cymba chonca. GammaCore is a transcervical, self-administered VNS device stimulating through the neck to the cervical VN (Yuan and Silberstein 2016)

TC-VNS (gammaCore, electroCore LLC, Basking Ridge, NJ, USA, Fig. 3) is likely to stimulate both afferent and efferent VN fibers in the carotid sheath and is approved by the FDA for the treatment of cluster headaches and migraines (Mertens et al. 2018). This TC-VNS device also obtained the CE marks for use in bronchoconstriction, primary headache, epilepsy, anxiety, depression, and gastric motility disorders (Mwamburi et al. 2017). No drug interactions or multidose-related adverse effects have been associated with the gammaCore device (Silberstein et al. 2016; Goadsby et al. 2018; Gaul et al. 2016). The gammaCore device has also been studied to treat hemicrania continua (Nesbitt et al. 2012), asthma (Steyn et al. 2013), and Sjorgen’s syndrome (Tarn et al. 2019) with all studies achieving success in small sample sizes. Currently, there are ongoing clinical trials of TC-VNS for the treatment of Raynaud’s phenomena (ClinicalTrials.gov. Bethesda (MD): National Library of Medicine (US) 2020a), dyspepsia and irritable bowel syndrome (ClinicalTrials.gov. Bethesda (MD): National Library of Medicine (US) 2020c), and pancreatitis (ClinicalTrials.gov. Bethesda (MD): National Library of Medicine (US) 2020e). Although no optimal stimulation configuration has been found yet, in general low-voltage self-regulated stimulation is applied with 1 ms pulse width, 25 Hz frequency approximately during 2 min, and can be repeated up to 12 times (Holle-Lee and Gaul 2016).

TA-VNS stimulates the auricular branch of the VN, which innervates the cavity of conchae and cymba conchae, which is the only area innervated by the auricular branch of the VN (Peuker and Filler 2002). The Cerbomed device called NEMOS (Erlangen, Germany, Fig. 3) uses an intra-auricular electrode to stimulate the afferent nerve fibers of the vagal auricular branch (Ellrich 2019) and received European clearance in 2011 for the treatment of epilepsy and depression in 2010, and for pain relief in 2012. This device is available in Austria, Germany, Italy, Switzerland, and the UK. The optimal stimulation is chosen individually by the patients based on the intensity to feel a non-painful stinging with a recommended stimulation duration up to 4 h per day. This individualization procedure hinders the establishment of an optimal stimulation configuration, but in general, amplitude is set around 1 mA according to the above-mentioned pain thresholds, frequency varies from 20 to 30 Hz, and pulse width in normally below 1 ms (He et al. 2013). Another company, Auri-Stim, has developed the NET-1000, NET-2000, which was approved by the FDA in 2006 for the treatment of depression, anxiety and insomnia, and NET-3000 (Denver, CO, USA). These devices provide a similar stimulation however, NET-3000 is the most advanced version that allows more flexibility in the selection of the stimulation parameters as it includes 15 pre-programmed stimulation modes.

As previously introduced, SLE shares many symptoms with other chronic diseases including joint pain, joint swelling and fatigue and weakness (Cojocaru et al. 2011). Studies of the clinical impact of non-invasive VNS in these diseases are therefore of remarkable interest for studying the safety and efficacy of nVNS in SLE. To begin, one study found that TA-VNS significantly increased heart rate variability and reduced sympathetic outflow in healthy individuals (Clancy et al. 2014). Recent studies of TA-VNS have shown success in the treatment of chronic pain (Napadow et al. 2012), perceived pain (Busch et al. 2013; Janner et al. 2018), and migraines (Zhang et al.Straube et al. 2015; Garcia et al. 2017). In a recent study in RA patients, TA-VNS using a vibrotactile device in contact with the cymba concha applied twice a day for two consecutive days reduced the amount of circulating proinflammatory cytokines such as TNF, IL-6 and IL-1 *β* when compared with control subjects, who were stimulated at the gastrocnemius (Addorisio et al. 2019). Interestingly, disease attenuation in RA patients persisted for up to 7 days. In a double-blind sham-controlled pilot study in 18 patients with SLE with musculoskeletal pain, TA-VNS resulted in a significant reduction of pain (83.3% of TA-VNS subjects vs 16.7% sham-stimulated subjects), fatigue (83.3% of TA-VNS subjects vs 0% sham-stimulated subjects) and joint scores after 5 and 12 days (Aranow et al. 2021). Stimulation consisted on 5 min of pulses of transcutaneous electrical nerve stimulation (30 Hz frequency, 300 *μ*s pulse width) applied at the left ear, or sham stimulation (same device removing the battery) for 4 consecutive days. Pulse amplitude was set to the maximum amount tolerated by each subject without feeling pain. An encouraging result in addition to the clinical outcomes observed during the stimulation period, with improvement being significantly correlated with the cumulative current received over the days, is that these improvements continued through day 12, suggesting the long-lasting magnitude of the effects. Moreover, no adverse side effects, such as headache, tinnitus or skin irritation, were reported during the study confirming its safety for the duration of this study. Currently, there are ongoing clinical trials using TA-VNS for the treatment of RA (ClinicalTrials.gov. Bethesda (MD): National Library of Medicine (US) 2020b), and other chronic diseases like pain perception (ClinicalTrials.gov. Bethesda (MD): National Library of Medicine (US) 2020d), depression and fibromyalgia (ClinicalTrials.gov. Bethesda (MD): National Library of Medicine (US) 2020f).

Table 2 shows the studies listed above using nVNS and the device configuration during the intervention. The gammaCore device using TC-VNS is seen to follow similar settings for most of the studies however, TA-VNS studies are irregular. Many of the studies included in Table 2 that utilize TA-VNS chose to use a combination of an electrode fitted to the ear and a current stimulator. Two studies used the Nemos device (Straube et al. 2015; Busch et al. 2013) and one study used an oscillatory vibrotactile device (Addorisio et al. 2019). The amplitude was generally set for each individual to a constant tingling, non-painful sensation. Interestingly, 25 Hz was the most frequently used frequency with other frequencies set to 5 kHz, 168Hz, 100 Hz, 30Hz, and 1 Hz. Only two studies have explored the low-frequency range to stimulate the vagal efferent fibres and thus, the CAP. Straube et al. (Straube et al. 2015) compared 25 Hz and 1 Hz in the treatment of chronic migraines and found a significantly larger reduction in headache days in the 1 Hz group. This finding warrants further research into the low-frequency stimulation of the VN via nVNS.

**Table 2:** Overview of non-invasive VNS configuration and therapeutic application

nVNS device	Condition	Waveform	Amplitude	Pulse width (ms)	Frequency (Hz)	Train duration
gammaCore (TC-VNS)	Cluster headache (Silberstein et al. 2016)	5kHz sine wave burst	24V/60mA	5x200 *μ*S (1ms)	25	120ms
gammaCore (TC-VNS)	Cluster headache (Goadsby et al. 2018)	-	24V	5x200 *μ*S (1ms)	5000	3x120s
gammaCore (TC-VNS)	Chronic headache (Gaul et al. 2016)	5kHz sine wave burst	Max. 24V/60mA	5x200 *μ*S (1ms)	25	3x120s (5min rest)
gammaCore (TC-VNS)	Healthy, neural activation validation (Nonis et al. 2017)	5kHz sine wave burst	6V-18 V, max. output 60 mA	5x200 *μ*S (1ms)	25	3x120s (5min rest)
gammaCore (TC-VNS)	Episodic & chronic migraines (Kinfe et al. 2015)	5kHz sine wave burst	0V-24V	5x200 *μ*S (1ms)	25	2x120s
gammaCore (TC-VNS)	Menstrual migraine (Grazzi et al. 2016)	5kHz sine wave burst	Max. 24V/60mA	5x200 *μ*S (1ms)	25	2x120s
gammaCore (TC-VNS)	Acute asthma (Steyn et al. 2013)	-	-	-	-	2x60s (30min rest)
gammaCore (TC-VNS)	Primary Sjögren’s syndrome (Tarn et al. 2019)	5kHz sine wave burst	Low voltage	5x200 *μ*S (1ms)	25	90s
Non-specific (TA-VNS)	Chronic Pelvic Pain (Napadow et al. 2012)	Rectangular pulses	Moderate to strong (non-painful) sensation	450 *μ*S (1ms)	30	0.5s
Nemos (TA-VNS)	Pain Perception (Busch et al. 2013)	Modified monophasic rectangle impulse	0.25 - 10 mA (constant tingling sensation)	250 *μ*S	25	-
Non-specific (TA-VNS)	Pain Perception (Janner et al. 2018)	Square impluses delivered in blocks of 9 impulses	Constant tingling sensation	2x200 *μ*S (s)	100	-
Non-specific (TA-VNS)	Migraine (Zhang et al.)	-	1.5-3mA	0.2ms	1	-
Nemos (TA-VNS)	Chronic Migraine (Straube et al. 2015)	-	Tingling, non-painful sensation	250 *μ*S	1/25	30s on, 30s off (20min)
Non-specific (TA-VNS)	Migraine (Garcia et al. 2017)	Rectangular pulses	Moderate to strong (non-painful) sensation	450 *μ*S	30	0.5s
Non-specific (TA-VNS)	Rheumatoid arthritis (Addorisio et al. 2019)	-	Horizontal: 0.008" Vertical: 0.005"	-	*∼*168	-

Abbreviations: nVNS, non-invasive vagus nerve stimulation; TC-VNS, transcutaneous cervical vagus nerve stimulation; TA-VNS, transcutaneous auricular vagus nerve stimulation

## Conclusions

There is no denying that a link exists between autonomic dysfunction and SLE. The ability to manipulate the VN, via electrical stimulation, to reduce inflammation in the long term and to maintain remission status for as long a period as possible is of great interest. Although, SLE has not been studied in great detail in terms of implantable VNS, other inflammatory conditions such as, sepsis, lung injury, diabetes, obesity, migraines, RA, fibromyalgia, and Crohn’s disease have shown promising pilot clinical trial results with studies for fibromyalgia and Crohn’s disease achieving remission in some patients. Similarly, in nVNS, hemicrania continua, menstrual migraine, gastroparesis, asthma, Sjorgen’s syndrome, chronic pelvic pain, pain perception, and RA have all shown positive results in small sample sizes with no significant serious device-related adverse events reported. These results are of great importance for SLE as there are many symptoms and clinical manifestations that are shared between SLE and these diseases and whose improvement and even remission are extremely encouraging and suggest that nVNS may be a viable treatment option for SLE (Cojocaru et al. 2011). In fact, one study dedicated to nVNS in SLE showed a significant reduction in pain, fatigue, and joint score (Aranow et al. 2021). All these trials motivate further research on the use of VNS and nVNS in multiple types of inflammatory diseases including SLE, where clinical studies continue to be carried out.

The study of nVNS for the treatment of SLE is in its infancy, which implies that there remains a lot to discover about nVNS and its application to SLE treatment, however, preliminary studies regarding the safety and efficacy of this new technological approach show great promise in this area (Aranow et al. 2021). Therefore, this review motivates the use of bioelectronic medicine through neuro-modulation as a promising new pathway to treat a number of serious chronic inflammatory diseases. The therapeutic impact of bioelectronic medicine can be boosted by replicating the body’s closed-loop mechanisms: metabolic and neuro-physiological biomarkers can be recorded and analyzed in real-time to accordingly adjust the characteristics of the electrical stimulation delivered to the peripheral nerves or directly to the organs to modulate their function (Güemes Gonzalez et al. 2020). Advances in bioelectronic medicine towards these closed-loop systems are supported by the development of new non-invasive or minimally invasive technology and algorithms, which enable a safe and effective interface with the nervous system.

## Data Availability

Not applicable. Declarations

## Abbreviations

ANS: Autonomic nervous system
CAP: Cholinergic anti-inflammatory pathway
HPA: Hypothalamus-pituitary-adrenal
IL: Interlukin
nVNS: Non-invasive vagus nerve stimulation
PNS: Parasympathetic nervous system
RA: Rheumatoid arthritis
SLE: Systemic lupus erythematosus
TA-VNS: Transcutaneous auricular vagus nerve stimulation
TC-VNS: Transcutaneous cervical vagus nerve stimulation
TNF: Tumor-necrosis factor
VN: Vagus nerve
VNS: Vagus nerve stimulation
